# A Foundation for Enterprise Imaging: HIMSS-SIIM Collaborative White Paper

**DOI:** 10.1007/s10278-016-9882-0

**Published:** 2016-05-31

**Authors:** Christopher J. Roth, Louis M. Lannum, Kenneth R. Persons

**Affiliations:** 1 Duke Health Technology Solutions, Hock Plaza, 2424 Erwin Road, Durham, NC 27705 USA; 2 Department of Radiology, Duke University Hospital, 2301 Erwin Road, Box 3808, Durham, NC 27710 USA; 3 Information Technology Division, Cleveland Clinic, 572 Lake Forest Dr., Bay Village, OH 44140-2513 USA; 4 Mayo Clinic IT, 200 3rd Ave SW, Pb 2-58, Rochester, MN 55905 USA

**Keywords:** Archive, Bioinformatics, PACS, Clinical image viewing, Diagnostic imaging, Digital Imaging and Communications in Medicine (DICOM), Electronic medical record (EMR), Enterprise imaging, Enterprise PACS, Imaging informatics, Imaging informatics administration, Multimedia, Systems integration, Image data, Image viewer

## Abstract

Care providers today routinely obtain valuable clinical multimedia with mobile devices, scope cameras, ultrasound, and many other modalities at the point of care. Image capture and storage workflows may be heterogeneous across an enterprise, and as a result, they often are not well incorporated in the electronic health record. Enterprise Imaging refers to a set of strategies, initiatives, and workflows implemented across a healthcare enterprise to consistently and optimally capture, index, manage, store, distribute, view, exchange, and analyze all clinical imaging and multimedia content to enhance the electronic health record. This paper is intended to introduce Enterprise Imaging as an important initiative to clinical and informatics leadership, and outline its key elements of governance, strategy, infrastructure, common multimedia content, acquisition workflows, enterprise image viewers, and image exchange services.

## Introduction

Images are captured in many traditional and emerging settings, and these images represent valuable care information that must be managed systematically and widely available. Having access to clinical imaging and multimedia content has been acknowledged as an important part of patient care and fully realizing the value of electronic health records (EHRs) [1, 2].

As EHRs were installed, enterprises learned that many care groups had similarly isolated and fragmented approaches to image management [3]. Enterprises also learned that many specialties had similar image capture, image storage, and image distribution unmet needs [4–6]. In many clinical specialties, such as dermatology or endoscopy, textual descriptions of an image often scream for the image itself; supplementing text with the images better conveys information. Enterprise Imaging as a program or initiative developed as a result of the unmet need to clinically support imaging workflows, procure and manage scalable IT infrastructure, develop operational policy, and centralize governance and communications to accommodate the full body of enterprise multimedia clinical content, the imaging of a health enterprise.

The term “Enterprise Imaging” is increasingly common parlance in the clinical and industry communities, though the exact definition has varied [7–9]. Despite the growing interest, a recent PubMed search for “enterprise imaging” uncovered only two generally unrelated resources [10]. The collaborative HIMSS-SIIM member workgroup on the Definition of Enterprise Imaging defines Enterprise Imaging as “a set of strategies, initiatives and workflows implemented across a healthcare enterprise to consistently and optimally capture, index, manage, store, distribute, view, exchange, and analyze all clinical imaging and multimedia content to enhance the electronic health record.”

## What Enterprise Imaging Encompasses

A successful Enterprise Imaging (EI) program encompasses a number of key elements, each of which will be briefly summarized in this introductory paper. Many of these topics will be discussed in more depth in upcoming HIMSS-SIIM collaboration white papers dedicated to the subject.

These key elements for a successful Enterprise Imaging program are as follows:

1. Governance
2. Enterprise Imaging Strategy
3. Enterprise Imaging Platform (Infrastructure)
4. Clinical Images and Multimedia Content
5. EHR Enterprise Viewer
6. Image exchange services
7. Image analytics

## Governance

Effective and engaged governance is key to a successful EI strategy implementation. The HIMSS-SIIM collaborative workgroup defines Enterprise Imaging governance as “the decision-making body, framework, and process to oversee and develop strategies for the enterprise imaging program, technology, information, clinical use, and available financial resources.” A dedicated HIMSS-SIIM white paper on EI governance is in press. [11]. Like non-imaging healthcare governance groups, the governance bodies for EI are responsible for bringing together a wide group of clinical, administrative, and information technology stakeholders to make decisions [12–15].

Governance must be addressed early in embarking on EI. When just starting, typically each department owns, maintains, and controls its own imaging operations, data governance, IT support personnel, and technology infrastructure. There is typically little cross-department knowledge or infrastructure sharing. Personal and professional culture between departments may be non-existent or even negative because of historical turf battles. It is critical to start anew with a core group of constructive stakeholders, executives, and sponsorship in overseeing all imaging activities and service lines.

## Enterprise Imaging Strategy

A thoughtful and on-paper strategic plan vetted through governance should make clear the needed EI infrastructure and services. A beginning strategy and roadmap will at least include plans for the aforementioned key elements (governance, the enterprise image management platform, content, content viewing, EHR integration, image exchange services, and analytics).

Enterprise Imaging requirements, scope, and outcome strategic decisions are particularly critical [16]. These decisions inform the suitability of any already owned technology for the EI platform and thus also make apparent capital or operational funding requirements to implement the program. They also make clear those applications, storage, and viewers that do not fit with long-term enterprise plans. Roadmaps to sunset these components should be considered early, objectively, and transparently to all stakeholders. A restructuring of IT support away from departmental approaches toward enterprise may be necessary, and may involve new support personnel, or reassignment or release of personnel with deep relationships and long track records. All of these contribute to timeline decisions, as EI cannot be implemented without the infrastructure that supports the departmental imaging workflows. The above are delicate and often contentious discussions where strong governance proves invaluable. Engaging the specialties creating images in the strategic decisions will become important when clinical champions become needed during infrastructure and application selection, workflow redesign, and implementation.

## Enterprise Imaging Platform

**Fig. 1:**
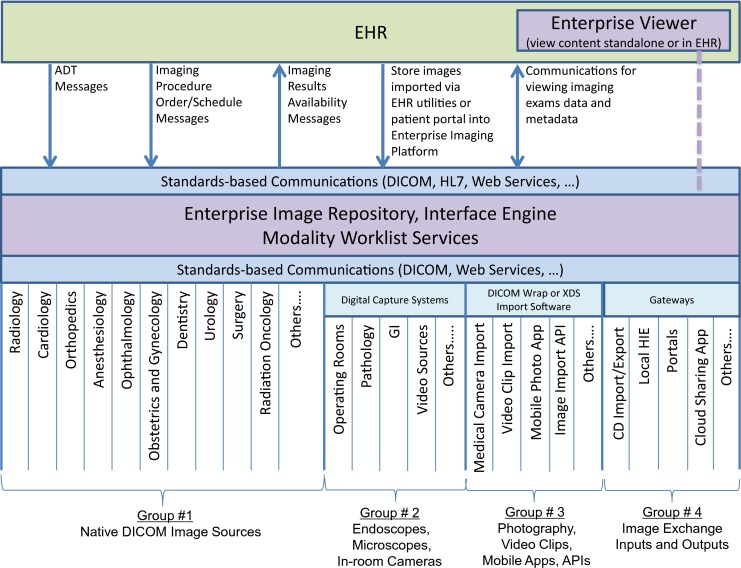
An Enterprise Imaging platform provides the standards-based, enterprise infrastructure to support departmental imaging workflows. This includes modality worklist services, image archival, index, enterprise viewer application viewing within or outside the EHR, query/retrieve of imaging content from most departments, as well as image exchange capabilities

The EI platform provides the infrastructure, modalities, devices, and integration points on which strategies can be based (Fig. 1). A standards-based DICOM and non-DICOM clinical image and video storage repository is central to the EI platform. This central repository may be a vendor neutral archive or, if it meets defined enterprise-wide requirements, an existing PACS archive. It includes an index of the image and meta-information content in the archive. The archive should be modality agnostic, modality vendor agnostic, specialty and service line agnostic, and viewer agnostic. Standards-based interfaces and communications, including DICOM, HL7, and standards-based Web Services, connect, enable, and support image acquisition workflows across modalities and departments [17–19]. Image acquisition devices that support these standards may store their images, with meta-information, into the VNA. Acquisition devices that are supported include departmental DICOM imaging modalities, point-of-care acquisition modalities, handheld device photo or video apps, digital capture systems in procedure rooms, image exchange gateways, and software designed to import content saved on a disk or received by referring or patient portals.

Core services of the platform include the following:

1. “Standards-based” integration, including provision of modality worklist services to provide accurate patient/procedure information for most departmental systems that create images in their workflows.
2. Providing a reliable archival service, with a patient-centric index, or registry of all content in the archive.
3. Provision of a thin- or zero-client Enterprise viewer that can display all images and multimedia content stored in the archive.
4. Support for standards-based access methods, such as DICOM, HL7, XDS/XDS-I. FHIR®, and DICOMweb™ services.
5. Support for various acquisition and import methods, such as native DICOM communications, DICOM “wrapping,” digital video capture systems, and exchange gateways.
6. EHR integration—including clinically relevant descriptors of imaging content, imaging orders created by the EHR where necessary, imaging results and availability notifications reported to the EHR, and image viewing from the EHR.
7. Providing reliable retrieval services, with secure access control and auditing.
8. Disaster recovery and business continuity with minimal to no disruption to service.

In the past, the primary role of an EHR in the imaging domain was to create and send orders to a Radiology Information System (IS) or PACS, to receive the final imaging results (the signed-off diagnostic report) and a link to view the images in the departmental PACS viewer. EHRs today commonly support “information system” services and workflows for radiology, cardiology, obstetrics, and other imaging departments.

As with other medical procedures, the EHR can use order information to index and track imaging procedures [20]. Some imaging procedure workflows are order based, while some are encounter based [21]. For encounter-based imaging procedures, the EI platform can either use an image availability notification to report “unsolicited” imaging results or it can request the EHR to create an order under which the imaging results can be associated. In this way, imaging procedures that are order based and those that are not order based can be notified, indexed, and made available in the EHR with clinically relevant descriptors.

## Clinical Imaging and Multimedia Content

Images serve many purposes in patient care, with many operational workflows driving their creation [22]. Compartmentalizing the spectrum of content routinely captured today is difficult. Categorizing and supporting content based on the medical specialty that performs the imaging has been the traditional model (Fig. 1). This works reasonably for diagnostic imaging because of consistency in operational workflow within departments. However, now ubiquitous handheld still image and video content does not fit well in this model because any provider with appropriate training and security access may store content in the EHR, sometimes bridging historical specialty lines. With increasing provider integration and graduate medical education programs requiring imaging competencies, even within the same enterprise, often many different specialties may perform nearly identical procedures; extremity radiography and bedside ultrasonography are examples of such procedures [23–27]. Images coming in from external entities are also hard to classify by specialty, as for patient convenience, they are stored commonly on the enterprise repository without a local specialist imager reviewing them. Finally, the traditional specialty-based model for grouping imaging content also does not reflect the depth, breadth, or purpose of images captured within each specialty or department.

For the purposes of classifying the various forms of EI content, using modalities alone as guides is similarly inadequate. Ultrasound images for example may take the form of diagnostic echocardiography, may be captured as part of a procedure such as a line placement, may be saved only as documentation of sonographic findings such as during a GI endoscopy, or may be integrated alongside structured and unstructured text as often is done in an ankle-brachial index vascular image-enriched clinical report. Differentiating imaging types using file structure, such as DICOM or JPEG, is also insufficient, as many forms of medical images not initially captured as DICOM are archived and viewed through DICOM communications. Categorizing by image consumer also will not work, as the same performed imaging exam may be used in many different ways; a single extremity radiograph may be consumed by radiologists for diagnosis, by primary care for referral, by orthopedic surgeons for operative planning, by revenue management during revenue justification, and by patients for social media.

**Fig. 2:**
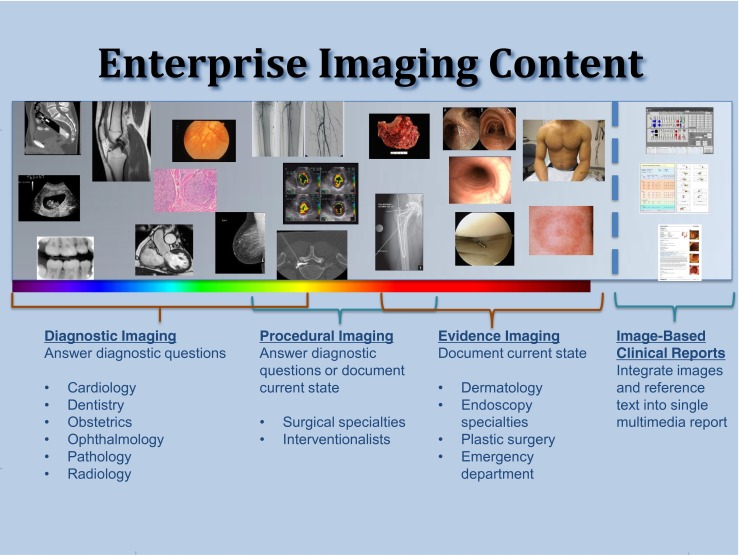
The broad spectrum of Enterprise Imaging content and common use cases

For educational and illustrative purposes, this HIMSS-SIIM Definition of Enterprise Imaging workgroup evaluated the above approaches and chose to describe and organize clinical imaging and multimedia content by the intent of its use by the performing providers (Fig. 2). This is to be distinguished from the imaging source (ultrasound modality, digital camera, etc.), the type of images created (DICOM Ultrasound, JPEG Photo, etc.), or the operational workflow that leads to the image(s). This approach defines content across a spectrum or continuum of four broad categories: diagnostic imaging, procedural imaging, evidence imaging, and image-based clinical reports. The framework is intended to be high level and educational, rather than provide hard and fast rules, as even within an individual encounter’s imaging examination, images often serve more than one intent and thus fall into more than one category. For example, during a cardiac catheterization, images may initially be captured and depict slow flow through a coronary vessel (diagnostic), as well as catheter and potentially stent manipulations and timestamps (procedural).

Nevertheless, categorizing the images into these four groups illustrates the complexity of solving EI for an organization. Such categorizing may help guide strategic decisions, as the strategies for image capture, storage, distribution, review, image intent, and value of the images tend to be more consistent within a category.

### Diagnostic Imaging

*What For:* Imaging obtained to elicit a differential diagnosis or confirm a clinical suspicion. Diagnostic images are usually obtained during a period of greater clinical uncertainty, where the etiology of patient symptoms is unknown and a broad scope of imaging may be necessary. Examples include the following:

- Routine 18-week obstetric ultrasound to diagnose congenital birth defects
- Carotid ultrasound after a patient experiences stroke symptoms
- Abdomen pelvis CT obtained in abdominal pain workup
- Ophthalmology optical coherence tomography for retinal evaluation in a glaucoma patient
- Echocardiography for possible valvular dysfunction
- Whole slide pathology imaging for surgical margin evaluation.

*Who:* “Traditional” diagnostic imaging specialties such as pathology, radiology, cardiology, ophthalmology, obstetrics, and dentistry.

*How:* These images often include an order-based workflow, diagnostic viewer image review, and subsequent interpretation documentation. A non-provider or provider obtains images according to standardized institutional protocols and modality techniques. A provider subsequently reviews the images to analyze the findings and answer diagnostic questions.

### Procedural Imaging

*What For:* Images are commonly obtained before, during, and after surgical and percutaneous invasive procedures. These images are intended to establish relevant procedure anatomy, guide a surgical approach, document timestamps of salient procedure events using modality-generated metadata, or confirm post-procedure conditions such as stent deployment. Often the images are simply “exhaust” from the performed procedure itself. After the procedure, the images serve as medicolegal or billing support and rarely provide new information to the performing specialty or other specialties. Examples include the following:

- Pre-operative suite orthopedic template or neurosurgical navigation images.
- Ultrasound images saved during an intensive care unit line placement specifically for support of billing.
- C-arm fluoroscopic images obtained to document needle localization and appropriate epidural contrast flow during percutaneous pain management procedures.
- Stereotactic breast images depicting a biopsy approach.
- CT fluoroscopic images captured during percutaneous abdominal fluid collection drain placement to guide trocar and pigtail catheter localization and deployment. Note again in this example, there are often minor components of diagnostic imaging (is there still a fluid collection?) and evidence imaging (did the collection get bigger?) to consider.

*Who:* Any specialty performing image-guided interventions, including many surgical subspecialties, cardiology, radiology, and other provider groups.

*How:* Generally the images are captured natively DICOM either with a dedicated associated result or the “result” found in the relevant procedure documentation.

### Evidence Imaging

*What For:* Evidence imaging includes images captured primarily for documentation of a patient’s current state. Like procedural imaging, these images often depict information already known to the performing specialty. Unlike procedural images, however, these images often provide significant clinical value after the initial encounter as the abnormalities are followed over time to evaluate treatment effects. Images often depict information of interest to specialties other than the one capturing the images, and frequently to patients themselves. The majority of evidence imaging use cases that do not require advanced image manipulation viewers. Examples include the following:

- Colonoscopic images to document appearance and location of polyps or ulcers
- Laryngoscopic images and video to document vocal cord paralysis or luminal lesions
- Arthroscopic images demonstrating cartilage irregularity
- Cutaneous visible light photography for tracking suspicious skin lesions, developing decubitus ulcers, or plastic surgery pre- and post-procedure states
- Gross pathology specimen prep images
- Short clinical audio-video files obtained for documenting functional gait, speech, and cranial nerve functional changes during or after cerebrovascular accident
- Emergency department visible light images of traumatic facial injuries
- Volume-rendered DICOM CT secondary captures of the same emergency department facial injuries and fractures, acting almost like a visible light photograph of DICOM data anatomy
- DICOM clinical trial or even “diagnostic” MRI brain images to follow tumor response to chemotherapy or immediate post neurosurgical residual tissue enhancement could possibly be classified as “evidence imaging”

*Who:* Specialties performing physical exams, endoscopic procedures, or sample analysis who want enduring documentation.

*How:* Evidence imaging is frequently (but not always) originally captured as visible light images using endoscopes and handheld or mobile device cameras, and may be saved in native (JPEG, MPEG, etc.), DICOM, or proprietary formats [28]. Encounter-based workflows are more common than order-based workflows for capturing of evidence imaging content [29].

### Image-Based Clinical Reports

*What For:* Increasingly, it is being recognized that concurrent delivery of images and textual information is more valuable than either alone [30–37]. Clinical departments and vendors are framing innovative ways to include imaging content referencing clinical text, and vice versa. Examples include the following:

- Colonoscopy image-enriched textual reports where thumbnail image depictions of lesions identified reference a colon schematic diagram for approximate physical localization. It can be difficult to describe the exact location of a lesion found on colonoscopy and images cross-referencing clinical text or a schematic diagram often best convey findings information.
- Vascular image-enriched textual reports longitudinally documenting an aneurysm initial size and appearance, subsequent stent placement, and later, endoleak.
- Some waveform documents stored as images for medical record integration, or alternatively stored as a scanned document.

*Who:* Image-based clinical reports are created by the specialty performing the procedure. They are made either for themselves at a future visit or as a customer service to the referring specialty.

*How:* Depending on their complexity, image-based clinical reports can be time intensive to create, given the potential needs for thumbnail image selection, text inclusion, cross referencing, formatting, and result distribution. Image-based clinical reports are generally loaded into the medical record. These reports may supplement or serve as the output or result of the encounter. Such reports that include images may be loaded through a document management system that also incorporates scanned clinical paperwork, such as consent forms, and even non-clinical paperwork. Thumbnail images stored via these mechanisms often are of poor quality when trying to extract them from the medical record for education and research development; it is usually preferable to save the full fidelity images in the VNA.

## EHR Enterprise Viewer Considerations

If an EHR today did not offer providers the ability to input their textual physical exam findings, or did not offer providers the ability to review the textual physical exam findings in another clinic, that EHR would be deemed incomplete and insufficient to meet the needs of the enterprise. Ironically, however, the images depicting physical exam states are commonly not available to providers when they are needed, leaving those EHRs similarly incomplete. At most sites, these images are not available in the EHR because an easy-to-use method of storage and viewing has not been deployed to providers.

One primary goal of an EI initiative is to deliver all forms of imaging to the electronic health record. An enterprise image viewer is necessary to achieve this. Widely distributing images in the EHR improves communication between providers because relevant images toward making a diagnosis are not siloed away from providers needing them [38]. It also may prove to decrease imaging utilization, as a redundant exam may not need to be performed if the original images are easily accessible [39]. Widely available and easily accessed imaging facilitates multidisciplinary learning by providers [40, 41]. With the increased responsibility patients are taking in their health care decisions, and the frequently high cost of imaging, patients are taking more interest in imaging performed; having those images available for consumption or download through patient portals will be expected [42].

Understanding image viewing needs (which vary just like image capture workflow needs) is important for the governance and leadership bodies to recognize in developing the EI strategy. A dedicated HIMSS-SIIM white paper on enterprise viewing is in press [43]. Most enterprise image viewing clinical users can be categorized into one of several groups:

1. Diagnostic image creators and interpreters needing the most advanced image manipulation and reporting capabilities
2. Surgical subspecialist providers also needing advanced image manipulation tools to plan a procedure
3. General provider and non-provider users needing access to basic image viewing tools
4. An external user such as a patient or referrer with a need for basic image review

Users in all four groups expect fast and efficient review and manipulation of image datasets on any desktop, laptop, or mobile device. The enterprise viewer will meet the needs of most image reviewers, though pre-surgical and diagnostic image review still typically requires a dedicated viewer with specialty-specific data integrations, functionality, calculations, and user interface for the most advanced patient care and provider efficiency.

## Image Exchange Services

Like clinical free text and structured data, images must be shared between providers and patients outside of the walls of the creating hospital or clinic. The EI platform should be well suited to provide both inbound and outbound image exchange services [44–48]. There are many sources of outside images brought into an enterprise, such as CD/DVD, health information exchanges (HIE), as well as patient portals, referring physician portals, and telehealth portals. These images are stored in the VNA to be indexed and viewed in the EHR, and are available to be pre-fetched onto PACS for comparison or secondary interpretation. For outbound image exchange, the EI platform can leverage the image repository to assemble the studies and reports for export either to a CD/DVD burner or to an electronic exchange gateway. Benefits to an enterprise-wide-level image exchange service include not only a single centralized operational model internally for export but also simplifying the training and operationalizing of outside entities to send inbound by providing a single receiving platform across specialties.

## Enabling Business and Clinical Analytics

Business and clinical reports associated with the acquisition and management of imaging content outside of radiology and cardiology are felt by the HIMSS-SIIM workgroups to be generally immature. Detailed analytical tools for EI are in their infancy [49, 50]. The EI platform provides an infrastructure that supplies both the image and associated metadata to enterprise data warehouses. Defining and standardizing this metadata across the enterprise provides a repository of data that can provide departments with detailed study statistics, utilization reports, and image acquisition patterns [51–54]. True deep learning and complex neural networks of image data contents are beginning to be developed that will play large roles in the future of EI [55–58].

## Conclusion

This HIMSS-SIIM collaborative white paper introduces the definition of Enterprise Imaging, the scope of what it includes, as well as the challenges and opportunities it presents. Subsequent white papers will delve further into the subject. Imaging is an important part of many care practices, as images are captured to answer a specific diagnostic question, plan or guide a surgical treatment, or document a procedure or finding. Understanding clinical multimedia content, as well as the workflows and methods involved in its acquisition and use, helps an organization define and design an enterprise-focused strategy to harness this relatively ignored volume of clinical data. A patient’s electronic health record should include easy access to the wide range of medical imaging records obtained for that patient across all departments and service lines. A well-defined, well thought-out strategy, with the right governance and infrastructure, will go a long way toward making that possible.
